# The relationship between T‐helper cell polarization and the RANKL/OPG ratio in gingival tissues from chronic periodontitis patients

**DOI:** 10.1002/cre2.192

**Published:** 2019-05-29

**Authors:** Chun‐Sheng Bi, Li‐Juan Sun, Hong‐Lei Qu, Fang Chen, Bei‐Min Tian, Fa‐Ming Chen

**Affiliations:** ^1^ State Key Laboratory of Military Stomatology and National Clinical Research Center for Oral Diseases, Department of Periodontology, School of Stomatology Fourth Military Medical University Xi'an China

**Keywords:** chronic periodontitis, T‐helper cell polarization, RANKL/OPG ratio, gingival tissue

## Abstract

This study aimed to investigate the relationship between inflammation‐related T‐helper cell polarization and the receptor activator for nuclear factor‐κB ligand (RANKL)/osteoprotegerin (OPG) ratio, which is associated with bone resorption or remodeling of chronic periodontitis patients. Gingival crevicular fluid (GCF) and gingival tissues were obtained from periodontally healthy individuals (PH group) and chronic periodontitis patients (CP group). The GCF levels of IFN‐γ, IL‐4, IL‐17, and IL‐10 linked to T‐helper cell polarization toward the Th1, Th2, Th17, and Treg phenotypes, respectively, were determined by ELISA. The expression levels of these cytokines and the polarized T‐helper cells in gingival tissues were assessed through immunohistochemical and immunofluorescence assays. In addition, the RANKL and OPG expression levels in gingival tissues were detected by immunohistochemical assays, and linear regression analysis was used to identify the potential relationship between T‐helper cell polarization and the RANKL/OPG ratio. In total, 22 individuals and 35 patients were enrolled in the present study. In both GCF and gingival tissues, increased levels of IL‐17 and the decreased levels of IL‐4 and IL‐10 were observed in the CP group. When polarized T‐helper cells were identified in gingival tissues, more Th1 and Th17 cells were found in the CP group, whereas more Th2 and Treg cells were found in the PH group. Although there was no significant difference in OPG expression between the two groups, the RANKL/OPG ratio in the CP group was higher than that in the PH group. The linear regression analysis showed that the presence of more Th1 and Th17 cells correlated with a higher RANKL/OPG ratio, whereas the presence of more Th2 cells correlated with a lower RANKL/OPG ratio. Th1 and Th17 cells are positively correlated and Th2 cells are negatively correlated with the RANKL/OPG ratio. Our data suggest that T‐helper cell polarization is closely linked to the RANKL/OPG ratio in gingival tissues from chronic periodontitis patients.

## 1 INTRODUCTION

Periodontitis, caused by altered host–biofilm interactions in the gingival crevice, is the most common infectious disease and leads to progressive alveolar bone destruction and eventual tooth loss in humans (Barbato et al., 2015; Papapanou & Susin, 2017). Therefore, periodontal bone resorption is closely linked to the host immune response to a microbial challenge (Díaz‐Zúñiga et al., 2017; Ghighi et al., 2018; Hienz, Paliwal, & Ivanovski, 2015; Pan et al., 2018; Takayanagi, 2005). During such pathological cascades, many immune cells polarize toward proinflammatory responses; for example, we previously reported increased levels of M1 phenotypes of macrophages in inflammatory gingival tissues (Zhou et al., 2019). Given the nature of the disease, it is essential to regulate the immune response involved in periodontal reparative events when designing new therapies for periodontitis (e.g., see Bi et al., 2019; He et al., 2019; Ni et al., 2019; Wu et al., 2019; Yu, Wu, Yin, & Chen, 2016). Periodontal pathogens also activate T cells, especially CD4+ T cells (T‐helper cells), to develop into various subsets and initiate the acquired immune response (Knight, Liu, Seymour, Faggion, & Cullinan, 2016; Silva et al., 2015); subsequently, a spectrum of proinflammatory cytokines are released, which induces tissue damage and bone destruction (Hienz et al., 2015). It is now recognized that CD4+ T cells also mediate host immune responses largely via the characteristic cytokines produced by polarized CD4+ T cells, including T‐helper 1 cells (Th1 cells), T‐helper 2 cells (Th2 cells), T‐helper 17 cells (Th17 cells), and regulatory T cells (Treg cells; Murphy & Reiner, 2002).

Generally, Th1 cell‐derived cytokines, such as interferon‐γ (IFN‐γ) and interleukin‐12 (IL‐12), are associated with postinfection tissue destruction in periodontitis, while Th2 cell‐derived cytokines, such as IL‐4, have been thought to be protective factors for the repair of bone loss (Queiroz‐Junior et al., 2010; Repeke et al., 2011). In recent years, new CD4+ T cell subsets have been identified and demonstrated to play important roles in regulating host responses; these subsets include but are not limited to Th17, Th9, and Treg cells, which have enriched the initial Th1/Th2 dichotomy paradigm and offered new insights into the relationship between host immune responses and periodontitis (Buduneli & Kinane, 2011; Jäger & Kuchroo, 2010; Takayanagi et al., 2000). For example, the Th17 subset causes bone destruction during osteolytic and infectious processes by secreting proinflammatory cytokines, such as IL‐17 and IL‐21 (Bettelli, Korn, & Kuchroo, 2007; Zhu & Paul, 2010), whereas Treg cells are useful for bone repair, not only by warding off infection and weakening bacterial virulence but also by diminishing host immune responses, which leads to the incomplete elimination of some foreign agents. Interestingly, the regulation of Th cell polarization either locally or systemically has been demonstrated to inhibit bone loss in experimental periodontitis (Bi et al., 2019). The involvement of Th17 and Treg subpopulations in periodontal disease makes the inflammatory scenario of periodontitis more complex; unfortunately, the protective or destructive roles of Th17 and Treg cells, in addition to Th1 and Th2 cells, within the overall framework associated with tissue destruction and disease progression remain largely unexplored (Dar, Azam, Anupam, Mondal, & Srivastava, 2018; Garlet, 2010).

Given that the local ratio of receptor activator for nuclear factor‐κB ligand (RANKL) to osteoprotegerin (OPG) in periodontal tissues has been an important hallmark for assessing the degree of bone resorption and the pathological process of periodontitis (Bi et al., 2019; Bostanci et al., 2007; Mogi, Otogoto, Ota, & Togari, 2004), we designed this study to investigate the relationship between inflammation‐related T‐helper cell polarization (e.g., Th1, Th2, Th17, and Treg polarization) and the RANKL/OPG ratio based on gingival crevicular fluid (GCF) and gingival tissues from periodontally healthy (PH) individuals and patients with advanced chronic periodontitis (CP). Our study will provide new clinical evidence concerning the nexus between the immune response and bone destruction during periodontal protective or destructive events.

## 2 MATERIAL AND METHODS

### 2.1 Study design and ethics

This investigation was a case‐controlled, cross‐sectional study. PH individuals with clinical attachment level (CAL) < 1 mm and without alveolar bone resorption on radiograph were assigned to the PH group, and patients with a probing depth > 6 mm, CAL > 4 mm, and bone absorption > half of the root length and who were diagnosed with severe CP according to the American Academy of Periodontology 1999 criteria were allocated to the CP group. GCF and gingival tissues from both groups were included to investigate how inflammation‐related T‐helper cell polarization is involved in periodontitis‐associated bone resorption. This study was approved by the Ethics Committee of Fourth Military Medical University. Subjects who visited the Department of Periodontology in the Stomatological Hospital of Fourth Military Medical University for tooth cleaning or periodontitis‐related reasons during a period of 7 months from March 2017 to September 2017 were asked to participate in this study.

### 2.2 Inclusion and exclusion criteria

Each participant involved in this study was required to have more than 20 teeth in his or her mouth. The exclusion criteria for the participants were as follows: (a) systemic disease affecting periodontal status (e.g., immunological disorders, diabetes mellitus, and AIDS); (b) a medical history of systemic antibiotic and nonsteroid anti‐inflammatory drug use during the previous 4 months for monitoring or treatment procedures (e.g., heart conditions and joint replacements); (c) pregnant or lactating, with nonsurgical periodontal treatment within the past 6 months or surgical periodontal treatment within the past 12 months; and (d) periapical inflammation, aggressive periodontitis, smoking, long‐term medication with contraceptive and similar hormone compounds, salivary gland diseases, or orthodontic treatment.

### 2.3 Enrollment of participants

The potential participants underwent a general oral examination, including bleeding on probing, probing depth, gingival recession, and CAL; those who were PH but had impacted mandibular third molars and those who had advanced CP were screened for this study. Then, the potential participants were informed of the purpose of this research, and those who agreed to participate were asked to provide written informed consent to contribute their GCF and gingival tissue (the gingival tissue biopsies were over 1 mm × 1 mm × 1 mm and obtained during periodontal surgery or tooth extraction) for subsequent investigations. The enrolled participants were divided into a PH group and a CP group.

### 2.4 Collection of GCF samples and gingival tissue biopsies

GCF samples and gingival tissue biopsies from the PH group were obtained from impacted mandibular third molars that were to be extracted, and samples from the CP group were obtained from teeth with severe periodontitis that were subjected to periodontal flap surgery, gingivectomy procedures, or tooth extraction. GCF samples were collected by PerioPaper Strips (Oraflow Inc., Smithtown, NY, USA) 2 weeks after supragingival scaling from the gingival sulcus between the distal gingival flap and mandibular third molar for the PH group and from the buccal aspect of mesial and distal sites of each selected tooth for the CP group. Before GCF was obtained from the gingival sulcus, the sampling tooth was dried by air syringe and isolated with cotton rolls to avoid contaminating the strips with saliva. Then, the strips were slowly inserted into the gingival sulcus or periodontal pockets along the surface of the tooth root until resistance was sensed for 30 s, avoiding blood contamination caused by any mechanical bruising to the soft tissues. After the samples were weighed, the weight of GCF absorbed by the strips was converted into the volume using a factor of 1 μl/μg, and the strips obtained from each subject were sealed in an Eppendorf tube with sufficient distilled water to dilute the sample by a factor of 100. Finally, all tubes were vortexed, centrifuged, and then stored at −80°C for further laboratory assays. For gingival biopsies, the samples were obtained during periodontal flap surgery, gingivectomy procedures, or tooth extraction. All harvested tissues were washed in saline, fixed in a 4% buffered formalin solution for paraffin embedding, and then subjected to immunohistochemistry and immunofluorescence assays.

### 2.5 Cytokine levels in GCF: ELISA

GCF samples were assayed to determine the levels of IFN‐γ, IL‐4, IL‐17, and IL‐10 using enzyme‐linked immunosorbent assay (ELISA) kits according to the manufacturer's instructions. Anti‐IFN‐γ (BMS228HS), anti‐IL‐4 (BMS225HS), and anti‐IL‐10 (BMS215HS) antibodies were obtained from eBioscience (San Diego, CA, USA), and an anti‐IL‐17 (BMS2017HS) antibody was obtained from BoYao (Shanghai, China). All ELISAs were repeated three times. Each standard curve was plotted according to the color intensity of the different standard antigen concentrations at 450 nm (BioTek, Winooski, VT, USA). Then, the concentrations of the four cytokines from all GCF samples were calculated according to standard curves and determined in picograms/ml (pg/ml). Finally, the original cytokine concentrations in the GCF were converted according to the dilution and determined in nanograms (ng/mL).

### 2.6 Immunohistochemical assay

Paraffin‐embedded gingival tissue was used to obtain serial histological sections (5 μm) for deparaffinization and hydration (using xylene and graded ethanol). After irrigation with deionized water, 0.3% hydrogen peroxide was applied to block endogenous peroxidase activity. Then, antigen retrieval was performed using heated ethylenediaminetetraacetic acid buffer to detect IL‐10, and citric acid buffer (pH 6.0) was used to detect other antigens (at 95°C for 20 min). Next, 4% goat serum in PBS (pH 7.4) was applied for serum blocking (at room temperature in a wet box device for 1 hr). After removing the serum blocking solution, all slides were incubated with primary antibodies directed against IFN‐γ (GB11107; Servicebio, Wuhan, China) in a 1:200 dilution, IL‐4 (ab239508; Abcam, Cambridge, UK) at a concentration of 5 μg/mL, IL‐17 (ab79056; Abcam) at a concentration of 5 μg/mL, IL‐10 (ab134742; Abcam) at a concentration of 5 μg/mL, OPG (ABC463; Millipore, Darmstadt, Germany) at a concentration of 5 μg/mL, or RANKL (HPA045142; Atlas Copco, Atlas, Sweden) in a 1:35 dilution (at 4°C overnight in a wet box device). After incubation with the primary antibodies, 100 μl of diluted biotinylated goat secondary antibody (matched with the primary antibody) was applied to the sections at room temperature in a wet box device for 1 hr. Subsequently, the sections were washed and incubated with streptavidin conjugated to horseradish peroxidase for 30 min at room temperature in a wet box device. Then, the slides were stained with diaminobenzidine according to the kit instructions (G1211; Servicebio, Wuhan, China) before counterstaining with hematoxylin. Meanwhile, an isotype control was performed using the matched isotype primary antibody, and a negative control was performed without the primary antibody. Finally, images were obtained with a TH4‐200 photo system (Olympus, Japan) and analyzed by ImageJ software (National Institutes of Health, Bethesda, MD, USA).

### 2.7 Immunofluorescence assays

For immunofluorescence assays, gingival slides were dewaxed and hydrated, and microwave antigen retrieval was performed in ethylenediaminetetraacetic acid buffer (pH 9.0). Following serum blocking (with 4% goat serum for 40 min), the sections were incubated with the following combinations of primary antibodies: anti‐CD4 (GB13064‐1; Servicebio, Wuhan, China) and anti‐IFN‐γ (ab9657; Abcam, Cambridge, UK), anti‐CD4 and anti‐IL‐4 (ab211212; Abcam), anti‐CD4 and anti‐IL‐10 (ab34843; Abcam), and anti‐CD4 and anti‐IL‐17 (ab79056; Abcam) in a 1:100 dilution overnight at 4°C. After primary antibody incubation, the slides were incubated with the following combination of secondary antibodies: Alexa Fluor 488‐conjugated goat anti‐mouse (ab150117; Abcam) and Alexa Fluor 647‐conjugated goat anti‐rabbit (ab150083; Abcam) for 50 min in the dark. Additionally, a control was performed by omitting the primary antibody. Then, the slides were stained with a 1:200 dilution of 4′‐6‐diamidino‐2‐phenylindole (DAPI) solution (Sigma‐Aldrich, Louis, MO, USA) for 10 min in the dark. Finally, the stained cells were visualized by fluorescence microscopy, and images were collected (NIKON ECLIPSE C1, Nikon DS‐U3, Japan).

### 2.8 Statistical analysis

All the experiments were executed three times, and the data were expressed as the mean ± standard deviation (*SD*) and analyzed using the SPSS 11.5 statistical package (SPSS Inc., Chicago, IL, USA). A chi‐square test was applied to test gender differences between the two groups. Because the sample did not conform with the assumption of a normal distribution, differences in the quantitative data between the two groups were detected by Mann–Whitney U tests. The Spearman correlation test was applied to evaluate the relationship between the RANKL/OPG ratio and Th1/Th2/Th17/Treg polarization. Statistical significance was indicated at a *p* value < .05.

## 3 RESULTS

### 3.1 Enrolled participants

**Figure 1:**
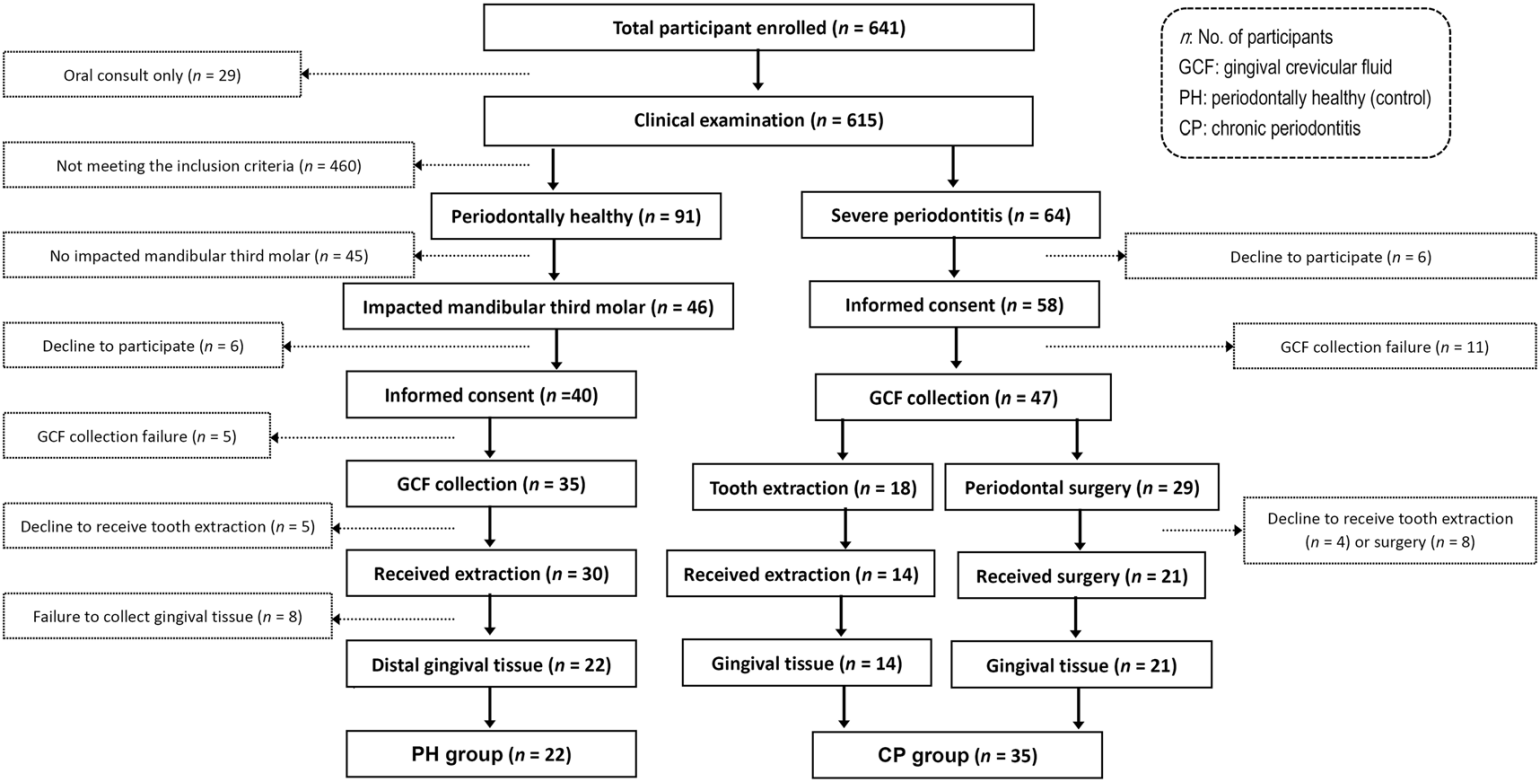
Flow diagram of the enrollment of the 57 participants in this study. CP, chronic periodontitis; GCF, gingival crevicular fluid; PH, periodontally healthy

A total of 57 subjects, including 22 PH individuals (PH group) and 35 chronic severe periodontitis patients, were enrolled in this study from the initial pool of 621 primary participants (Figure 1). GCF and gingival tissues were successfully obtained from these enrolled participants, and their clinical parameters are shown in Table 1.

**Table 1:** Clinical parameters of the participants

Group	PH	CP
Number of participants	22	35
Age (years)^a^	37.2 ± 8.7	41.5 ± 10.8
Gender (male/female)	10/12	19/16
Bleeding on probing (%)^a^	NA	76 ± 8.9
Gingival recession (mm)^a^	NA	1.85 ± 0.62
Probing depth (mm)^a^	2.4 ± 0.81	6.5 ± 1.17*
Clinical attachment level (mm)^a^	NA	5.4 ± 1.03

Abbreviations: PH, periodontally healthy group; CP, chronic periodontitis group.

a Data are shown as the mean ± standard deviation.

*

### 3.2 RANKL and OPG in gingival tissue

**Figure 2:**
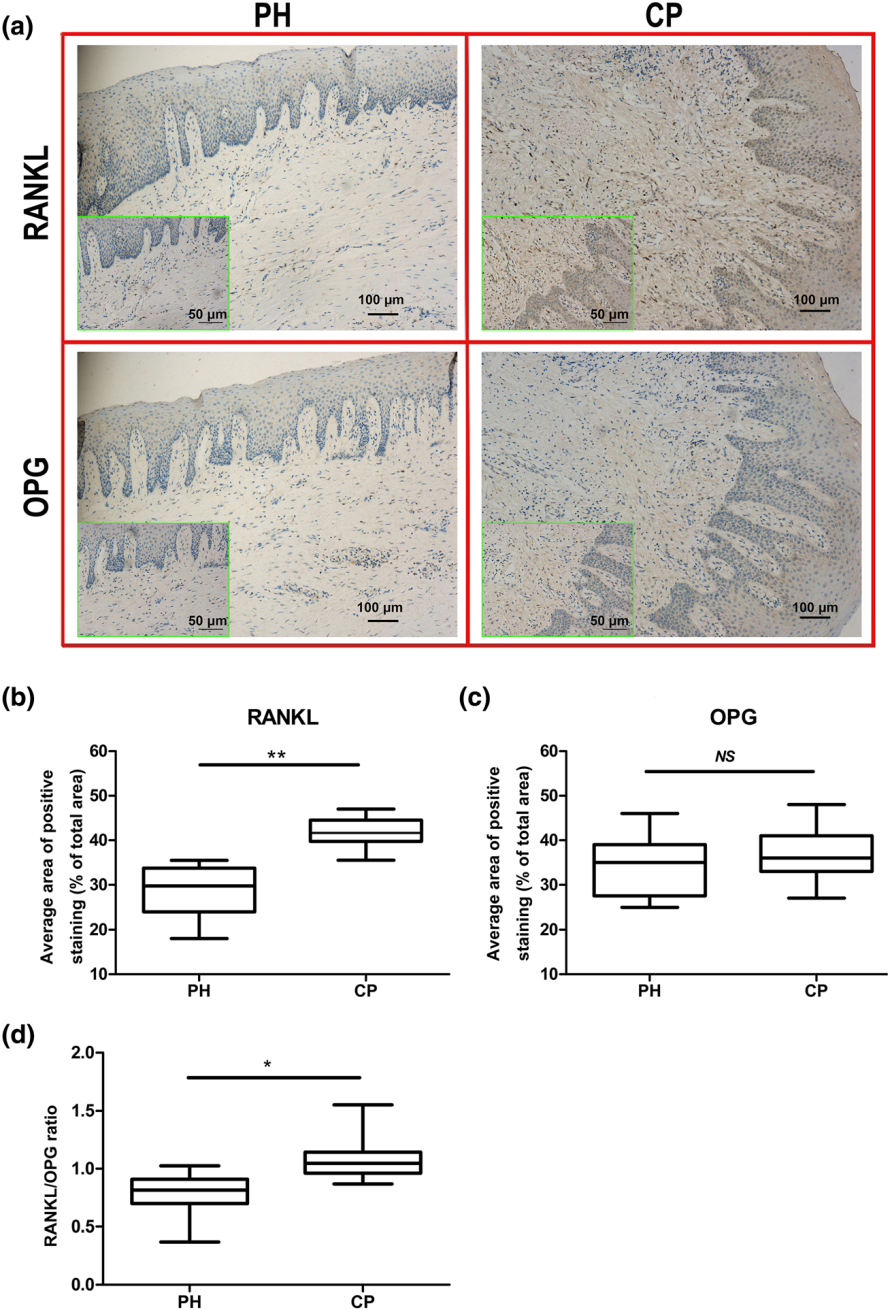
Receptor activator for nuclear factor‐κB ligand (RANKL) and osteoprotegerin (OPG) expression in the two groups (PH: *n* = 22, CP: *n* = 35). Brown areas represent the stained target protein. (a) RANKL and OPG expression in the two groups at 100× and 200× magnifications (green box). (b) After RANKL staining, a significant difference in the average area of positive staining (as a percentage of the total area) was observed between the two groups. (c) After OPG staining, no significant difference in the average area of positive staining (as a percentage of the total area) was observed between the two groups. (d) A significant difference in the RANKL/OPG ratio was observed between the two groups based on the average area of positive staining (as a percentage of the total area). ^**^ *p* < .01 and ^*^ *p* < .05 indicate significant differences between the periodontally healthy (PH) group and the chronic periodontitis (CP) group. ^NS^ *p* > .05 indicates that there is no significant difference between the two groups

RANKL and OPG in the gingival samples were detected by immunohistochemical staining, in which target proteins in the cells and tissues, including epithelial tissues and deeper connective tissues, were stained brown (Figure 2a). There were significantly more RANKL‐positive tissues in the CP group than in the PH group (*p* < .01; Figure 2b). In terms of OPG staining, however, there was no significant difference between the two groups (*p* > .05; Figure 2c). However, the RANKL/OPG ratio in the CP group (1.02 ± 0.33) was higher than that in the PH group (0.88 ± 0.24; *p* < .05; Figure 2d).

### 3.3 T‐helper cell‐related cytokine levels in GCF

**Figure 3:**
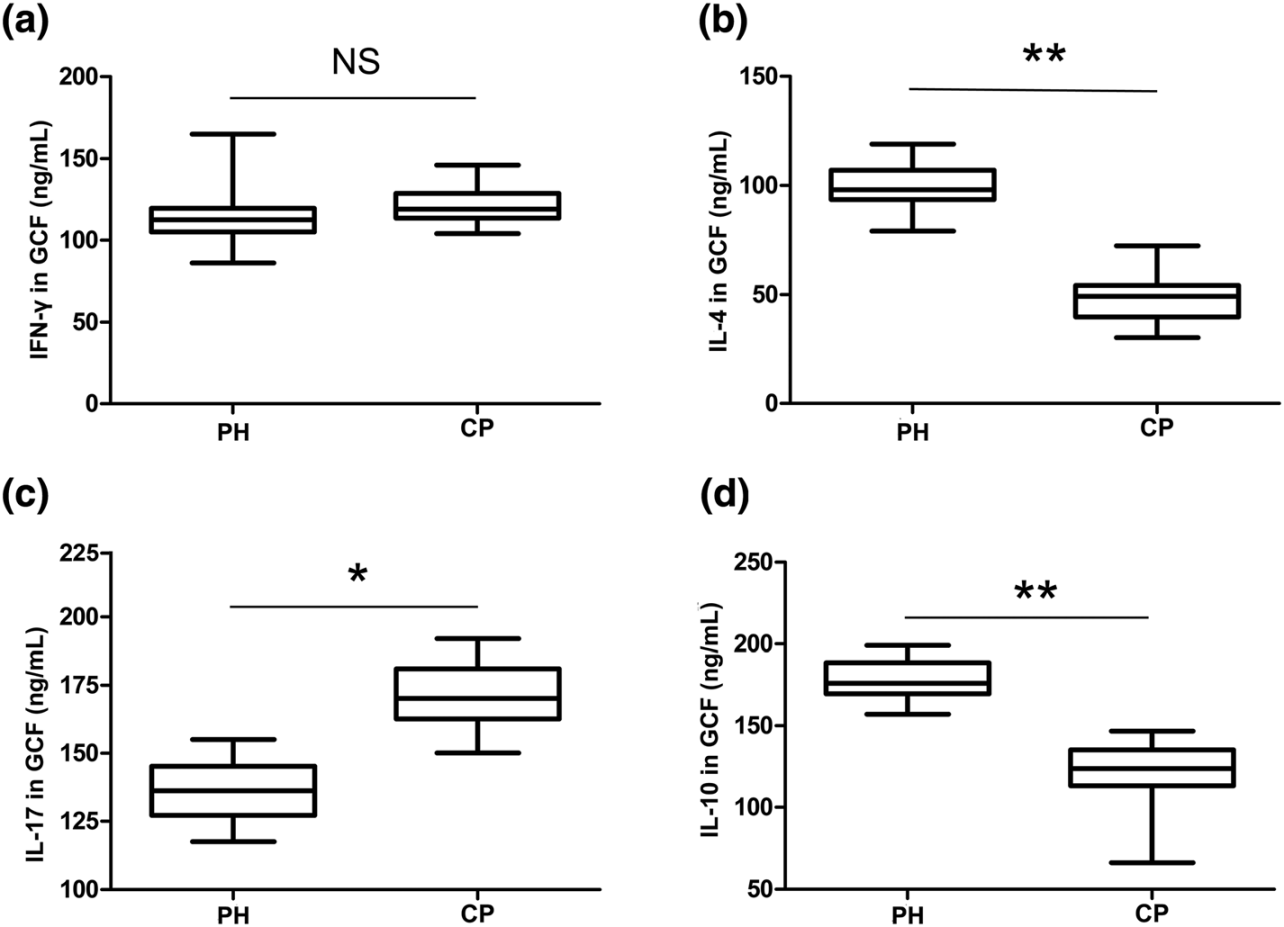
Cytokine levels of (a) IFN‐γ, (b) IL‐4, (c) IL‐17, and (d) IL‐10 in gingival crevicular fluid. ^**^ *p* < .01 and ^*^ *p* < .05 indicate significant differences between the periodontally healthy (PH) group (*n* = 22) and the chronic periodontitis (CP) group (*n* = 35). ^NS^ *p* > .05 indicates that there is no significant difference between the two groups

The levels of T‐helper cell‐related cytokines in GCF samples from all participants were measured by ELISA. In contrast to the levels of IFN‐γ, the levels of IL‐4, IL‐17, and IL‐10 exhibited significant differences between the PH group and the CP group (Figure 3). The IL‐4 and IL‐10 levels in the PH group were significantly higher than those in the CP group (Figure 3b,d), whereas the IL‐17 level in the CP group was higher than that in the PH group (Figure 3c).

### 3.4 T‐helper cell‐related cytokine levels in gingival tissue

**Figure 4:**
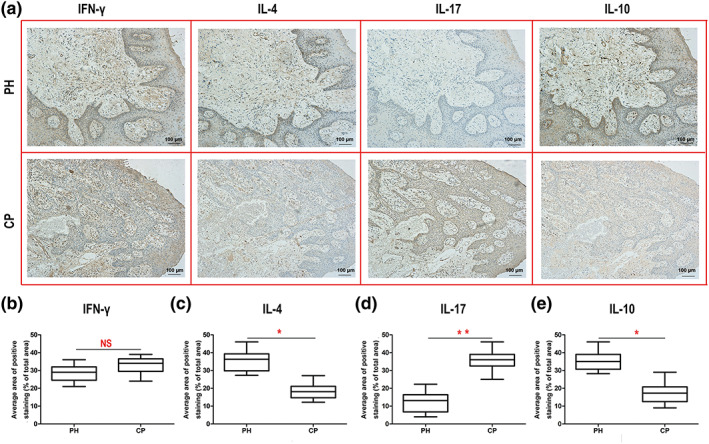
Immunohistochemistry image and analysis of each cytokine in gingival tissues of the two groups (periodontally healthy [PH]: *n* = 22, chronic periodontitis [CP]: *n* = 35). (a) The top four representative immunohistochemistry images (400×) are from healthy gingival tissue, and the bottom four are from the CP group. The brown‐stained area in each image is targeted to the indicated cytokine. Cytokine levels of (b) IFN‐γ, (c) IL‐4, (d) IL‐17, and (e) IL‐10 in gingival tissues. ^**^ *p* < .01 and ^*^ *p* < .05 indicate significant differences between the PH group and the CP group. ^NS^ *p* > .05 indicates that there is no significant difference between the two groups

The T‐helper cell‐related cytokines in gingival tissues were evaluated by immunohistochemistry assay (Figure 4a). As observed for the cytokine levels in GCF, the difference in IFN‐γ levels between the CP group and the PH group did not reach statistical significance (Figure 4b). However, the levels of IL‐4 and IL‐10 in the PH group were higher than those in the CP group (Figure 4c,e). Compared with the PH group, however, the CP group exhibited a higher level of IL‐17 (Figure 4d).

### 3.5 Th1/Th2/Th17/Treg cells in gingival tissue

**Figure 5:**
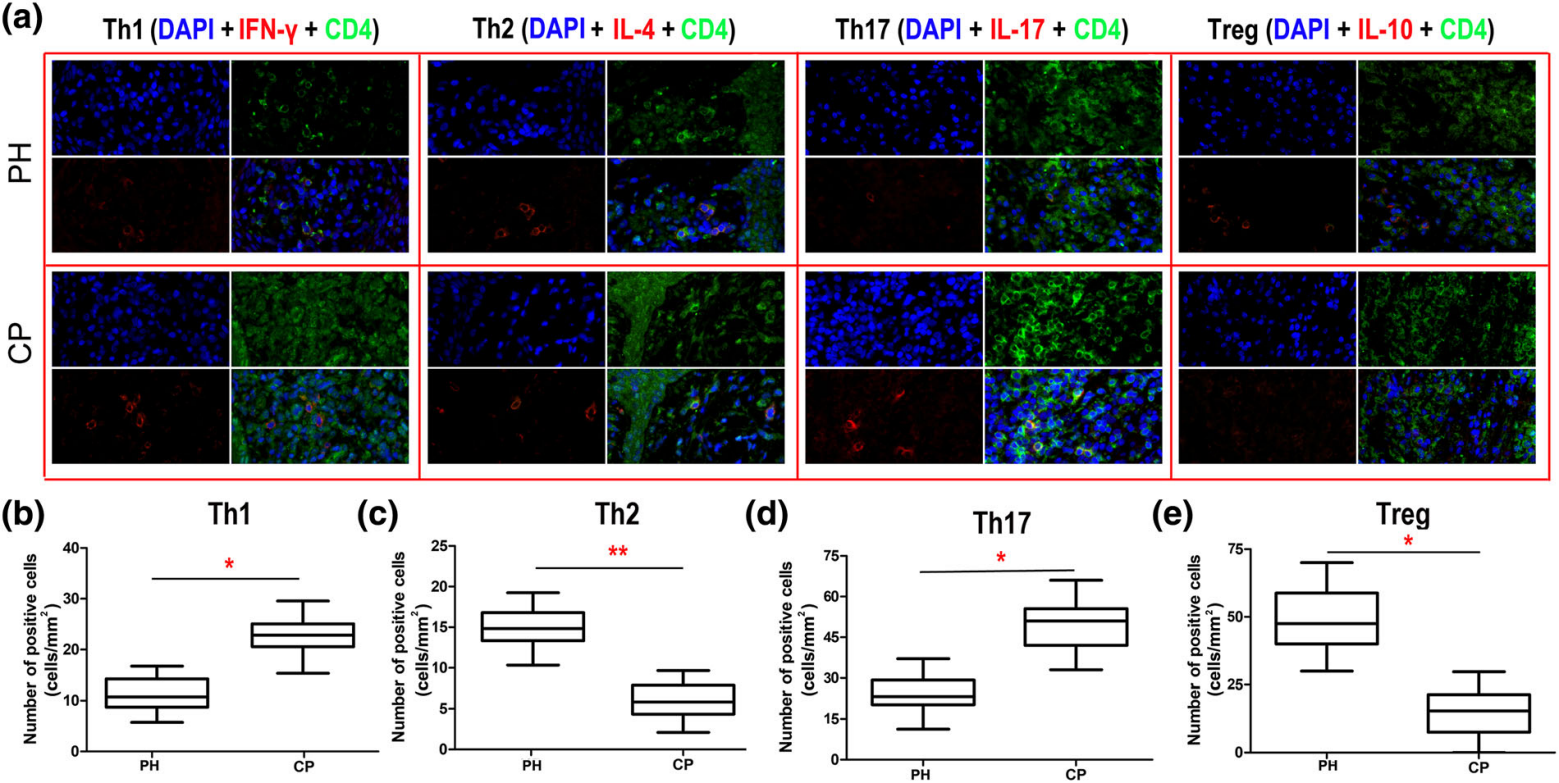
Immunofluorescence assay for Th1/Th2/Th17/Treg cells in gingival tissue of the two groups (periodontally healthy [PH]: *n* = 22, chronic periodontitis [CP]: *n* = 35). (a) The top four immunofluorescence images are from healthy gingival tissue, and the bottom four images are from the CP group. CD4 is marked by green fluorescence, the indicated proteins are marked by red fluorescence, and the cell nucleus is marked by blue fluorescence. Polarization levels of (b) Th1, (c) Th2, (d) Th17, and (e) Treg in gingival tissues. ^**^ *p* < .01 and ^*^ *p* < .05 indicate significant differences between the PH group and the CP group. ^NS^ *p* > .05 indicates that there is no significant difference between the two groups

Polarized Th1/Th2/Th17/Treg cells in the PH and CP groups were stained by immunofluorescence (green fluorescence for CD4, red fluorescence for each indicated protein, and blue fluorescence for the nucleus; Figure 5a). More Th1 and Th17 cells were found in the CP group than in the PH group (Figure 5b,d), but more Th2 and Treg cells were found in the PH group than in the CP group (Figure 5c,e).

### 3.6 Relationship between the RANKL/OPG ratio and Th1/Th2/Th17/Treg polarization

**Figure 6:**
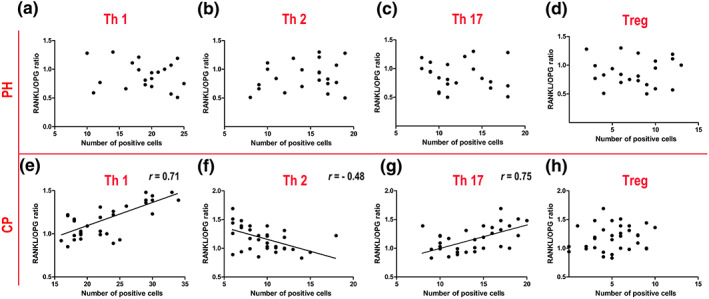
The relationship between the receptor activator for nuclear factor‐κB ligand (RANKL)/osteoprotegerin (OPG) ratio and Th1/Th2/Th17/Treg polarization. (a–d) Nonlinear relationships between the RANKL/OPG ratio and Th1/Th2/Th17/Treg polarization were observed in the periodontally healthy (PH) group (*n* = 22; *p* > .05). (e–g) Linear relationships between the RANKL/OPG ratio and Th1/Th2/Th17 polarization were observed in the chronic periodontitis (CP) group (*n* = 35; *p* < .05). (h) A nonlinear relationship between the RANKL/OPG ratio and Treg polarization was observed in gingival crevicular fluid from the CP group (*n* = 35; *p* > .05)

When the relationship between the RANKL/OPG ratio and Th1/Th2/Th17/Treg polarization was probed by linear regression analysis, no linear relationship between the RANKL/OPG ratio and Th1/Th2/Th17/Treg polarization was found in the PH group (*p* > .05; Figure 6a–d). In the CP group, a positive relationship between the RANKL/OPG ratio and Th1 and Th17 polarization was found in the linear regression, with regression coefficients of 0.71 and 0.76, respectively (*p* < .05; Figure 6e,g). In addition, a negative relationship between the RANKL/OPG ratio and Th2 polarization was detected in the linear regression (Figure 6f). Similar to the PH group, there was no linear relationship between the RANKL/OPG ratio and Treg polarization in the CP group (*p* > .05; Figure 6h).

## 4 DISCUSSION

This study aimed to investigate the clinical evidence related to the interaction between the immune response and alveolar bone resorption by evaluating the relationship between Th1/Th2/Th17/Treg polarization and the RANKL/OPG ratio. It is widely accepted that bone loss and remodeling are largely modulated by RANKL/OPG (Kawai et al., 2006). RANKL has the ability to induce the differentiation of preosteoclasts into mature osteoclasts by combining with RANK on the surface of preosteoclasts (Teitelbaum & Ross, 2003). However, this progression could be blocked by the high level of OPG, a localized decoy receptor for RANKL (Liu, Lerner, & Teng, 2010). Collectively, no significant difference in OPG expression was found between the two groups, which is not consistent with some previous reports (Oliveira et al., 2015; Ozaki et al., 2017), but both higher RANKL levels and higher RANKL/OPG ratios in gingival tissues in the CP group suggested that progressive bone resorption occurs in severe periodontitis. The presence of *Porphyromonas gingivalis* can increase the RANKL/OPG ratio at periodontitis sites (Belibasakis, Meier, Guggenheim, & Bostanci, 2011; Sakellari, Menti, & Konstantinidis, 2008), and a high RANKL/OPG ratio can facilitate inorganic matrix dissolution by cathepsin‐K and metalloprotease (Bostanci et al., 2008; Mogi & Otogoto, 2007). Therefore, the local RANKL/OPG ratio in periodontal tissues has been applied as an important hallmark for assessing the degree of bone resorption (Bostanci et al., 2007; Mogi et al., 2004). To explore the relationship between T‐helper cell polarization and alveolar bone loss, a linear regression analysis was applied to analyze the relevance between T‐helper cell polarization and the RANKL/OPG ratio in our study.

The levels of T‐helper cell polarization‐related cytokines were evaluated by ELISA in GCF and by immunohistochemical assay in gingival tissues; these cytokines can be used as specific indicators of immune responses and represent crucial secreted factors for regulatory immune responses at sites of progressive periodontal tissue lesions (Champagne et al., 2003; Lamster, 1992). In our study, a higher level of IL‐17 and lower levels of IL‐4 and IL‐10 in the CP group indicated potentially strong polarization toward Th17 cells and reduced levels of Th2 and Treg polarization in an inflammatory environment. Previous reports also detected increased expression of IL‐17 and decreased expression levels of IL‐4 and IL‐10 in GCF from periodontal patients (Mitani et al., 2015; Stadler et al., 2016). However, no significant difference in IFN‐γ was detected between the two groups (*p* > .05), which differs from the collective evidence that GCF IFN‐γ levels are significantly higher in CP patients, especially at severe clinical attachment loss sites (Garlet, 2010). As expected, the outcomes of immunohistochemical assays performed to detect related cytokine levels in gingival tissues were similar to the outcomes obtained for cytokine levels in GCF.

The levels of immune cell‐derived cytokines in GCF and gingival tissues are a consequence of a multitude of immune factors, which might reflect the tendency of CD4+ T cell polarization but may not indicate the precise level of CD4+ T cell polarization in gingival tissues. Therefore, further immunofluorescence assays were performed to evaluate the polarization level of CD4+ T cells. Our results showed a higher level of Th17 polarization and lower levels of Th2 and Treg polarization in the CP group, as evidenced by the levels of the cytokines IL‐4, IL‐17, and IL‐10. Unexpectedly, a higher level of Th1 polarization in the CP group was inconsistent with the level of IFN‐γ in GCF and gingival tissues between the two groups. It seems that IFN‐γ is not only released in response to T‐helper polarization but also produced by other immune cell populations. The Th1/Th2 balance is a crucial parameter for regulating host immune responses during CP. Th1 cells are capable of not only eliminating alien invaders but also inducing the local lesion in a host by releasing proinflammatory cytokines or damage products, such as IFN‐γ and RANKL (Dutzan et al., 2009). Several reports indicate that high concentrations of IFN‐γ in GCF are crucial for periodontal destruction, especially in alveolar lesions (Gao et al., 2007). In our findings, although there was no significant difference in IFN‐γ levels between the two groups, immunofluorescence assays indicated that higher levels of Th1 occurred in the CP group than in the PH group. Significantly, further investigation revealed a positive linear relationship between Th1 polarization and the RANKL/OPG ratio in the CP group, which indicated that a high level of Th1 polarization could aggravate alveolar bone resorption. Some evidence has provided a preliminary basis for understanding how IFN‐γ influences preosteoclast proliferation and maturation by regulating the levels of RANKL, tumor necrosis factor‐α (TNF‐α), and IL‐1β in vivo (Gao et al., 2007; Garlet et al., 2008). Besides, bacterial infection has a strong capacity to trigger Th1 and Th17 responses and associated cytokine production, which elicits alveolar bone resorption by upregulating RANKL expression during periodontitis (Monasterio et al., 2018).

In contrast, the lower levels of IL‐4 and Th2 in the CP group demonstrated that when the Th1/Th2 balance is disturbed in periodontitis patients, the proinflammatory Th1 cell role was promoted, whereas the anti‐inflammatory Th2 cell role was inhibited. Further, the significant negative correlation between Th2 polarization and the RANKL/OPG ration in the CP group also implied that a deficiency of the Th2 protective role could be linked to bone loss. Th2 responses have been considered a characteristic safeguard in periodontal tissues and can alleviate the severity of periodontal destruction by inducing complementary protective proteins, such as IL‐10 (Pestka et al., 2004; Pradeep, Roopa, & Swati, 2008). In previous studies, IL‐10 was regarded as a signature Th2 cytokine, but it has been reported to be closely tied to Treg cells in more recent studies (Pestka et al., 2004). The protective role of the Th2 response is instrumental for determining the RANKL/OPG ratio and the ratio of matrix metalloproteinase to tissue inhibitor of matrix metalloproteinases in periodontal tissues, which plays a key role in inhibiting bone loss (Garlet, Martins, Fonseca, Ferreira, & Silva, 2004; Ihn, Yamane, Asano, Kubo, & Tamaki, 2002). Indeed, the high level of IL‐4 from Th2 cells can suppress RANKL‐induced bone resorption and induce the rapid degradation of RANKL via the downregulation of the NF‐κB pathway after lipopolysaccharide‐induced NF‐κB activation in vitro. Therefore, the amplification of Th2 responses plays a protective role, and stimulation of IL‐4 can be viewed as an important target for the inhibition of bone resorption in CP.

Th17 cells, another subpopulation of T‐helper cells, were recently identified, and the presence of Th17 cells and IL‐17 is associated with disease severity and resultant bone destruction in periodontal lesions (Johnson, Wood, & Serio, 2004). In our study, higher levels of Th17 polarization and IL‐17 indicated the effect of Th17 on tissue destruction. Significantly, a positive linear relationship between Th17 polarization and the RANKL/OPG ratio in the CP group indicated that Th17 cells could be a key factor in bone lesions during periodontitis. It has been reported that more infiltrating Th17 cells and an accompanying higher level of IL‐17 were found in inflammatory gingival tissues, which demonstrates the destructive effect of Th17 polarization during CP (Cardoso et al., 2009). According to existing evidence, Th17 cells are responsible for tissue lesions by recruiting macrophages and neutrophils to inflamed periodontal tissues and inducing destructive products, including TNF‐α, IL‐6, IL‐8, prostaglandin 2, and IL‐1β (Silva et al., 2015). In particular, IL‐6 cannot only inhibit reparative cytokine generation but also accelerate Th17 cell differentiation to aggravate inflammatory reactions (Zheng et al., 2014). Moreover, inflammatory infiltrates can enhance Th17 levels and further upregulate RANKL expression via NF‐κB activity, and high Th17 levels are associated with high levels of alveolar bone resorption and low bone density (Kim, Kim, Kim, Cho, & Lee, 2015; Ribeiro et al., 2017).

Previous studies have verified that the deletion of Treg cells can cause abnormal immune regulation. Our data showed lower levels of IL‐10 and fewer Treg cells in gingival tissues in the CP group compared with those in the PH group. The expression level of IL‐10 is still controversial in periodontitis. Several studies have also suggested that upregulated IL‐10 and TGF‐β are found in periodontitis but IL‐17 was downregulated in the PH group compared with the CP group. However, not all studies are consistent with our findings, and some reports are even antithetical, with higher levels of IL‐10 found in infected gingival tissues than in healthy tissues (Dai, Bi, Lin, & Qi, 2017). Despite this controversy, IL‐10 has been regarded as an important biomarker for the Treg response. Our data did not show a correlation between the RANKL/OPG ratio and Treg polarization, although the level of IL‐10 was higher in the PH group than in the CP group. The protective role and immunologic self‐tolerance of Treg cells exert critical effects on tissue repair and reconstruction (Lee et al., 2016; Sakaguchi, 2004). FoxP3, another important biomarker of Treg cells, has been regarded as an inhibitor of Th17 differentiation via combination with retinoic acid receptor‐related orphan receptor‐γt (RORγt). The available evidence suggests that low levels of IL‐10 and FoxP3 occur during active periodontal destruction versus the high level observed during inactive destruction (Dutzan, Gamonal, Silva, Sanz, & Vernal, 2009; Hong, Williams, Jin, & Pike, 2000). However, bone lesions and the level of RANKL were negatively correlated with Treg cells in a rat model (Xiao et al., 2015). Indeed, the effect of IL‐10 on RANKL remains unclear. Some results even indicated that IL‐10 could upregulate RANKL (Wang, Guan, Jin, Lin, & Gao, 2015). Perhaps Treg cells play a role in bone resorption by regulating RANKL during the immune response, but IL‐10 is not an accurate biomarker for Treg cells. Therefore, it is critical to seek a representative biomarker for Treg cells and to clarify the mechanism by which Treg cells regulate RANKL.

## 5 CONCLUSIONS

Our results indicated that increased polarization of T‐helper cells toward Th1 and Th17 cells could enhance alveolar resorption by increasing the release of related cytokines, and the polarization of T‐helper cells toward Th2 cells was inhibited in association with a declining protective role for bone remodeling in CP. Therefore, a deeper understanding of the mechanism by which T‐helper cell polarization effects alveolar bone resorption could provide new avenues for periodontal immunotherapy.

## CONFLICT OF INTEREST

None declared.

## ETHICAL APPROVAL

This study was approved by the Ethics Committee of the Fourth Military Medical University.

## INFORMED CONSENT

All individuals who agreed to participate were asked to sign an informed consent form for the use of their GCF and gingival tissue (if available) for subsequent investigations.

## Footnotes

*
*p* < .01 when compared with the PH group.

## References

[cre2192-bib-0001] Barbato, L. , Francioni, E. , Bianchi, M. , Mascitelli, E. , Marco, L. B. , & Tonelli, D. P. (2015). Periodontitis and bone metabolism). Clinical Cases in Mineral and Bone Metabolism. The Official Journal of the Italian Society of Osteoporosis, Mineral Metabolism, and Skeletal Diseases, 12(2), 174–177. 10.11138/ccmbm/2015.12.2.174 PMC4625776 26604945

[cre2192-bib-0033] Belibasakis, G. N. , Meier, A. , Guggenheim, B. , & Bostanci, N. (2011). The RANKL–OPG system is differentially regulated by supragingival and subgingival biofilm supernatants. Cytokine, 55(1), 98–103. 10.1016/j.cyto.2011.03.009 21474331

[cre2192-bib-0022] Bettelli, E. , Korn, T. , & Kuchroo, V. K. (2007). Th17: The third member of the effector T cell trilogy. Current Opinion in Immunology, 19(6), 652–657. 10.1016/j.coi.2007.07.020 17766098 PMC2288775

[cre2192-bib-0009] Bi, C. S. , Wang, J. , Qu, H. L. , Li, X. , Tian, B. M. , Ge, S. , & Chen, F. M. (2019). Calcitriol suppresses LPS‐induced alveolar bone damage in rats by regulating T helper cell subset polarization. Journal of Periodontal Research. Epub ahead of print.. 10.1111/jre.12661 31095745

[cre2192-bib-0035] Bostanci, N. , Emingil, G. , Afacan, B. , Han, B. , Ilgenli, T. , Atilla, G. , … Belibasakis, G. N. (2008). Tumor necrosis factor‐α‐converting enzyme (TACE) levels in periodontal diseases. Journal of Dental Research, 87(3), 273–277. 10.1177/154405910808700311 18296613

[cre2192-bib-0026] Bostanci, N. , İlgenli, T. , Emingil, G. , Afacan, B. , Han, B. , Töz, H. , … Belibasakis, G. N. (2007). Gingival crevicular fluid levels of RANKL and OPG in periodontal diseases: Implications of their relative ratio. Journal of Clinical Periodontology, 34(5), 370–376. 10.1111/j.1600-051x.2007.01061.x 17355365

[cre2192-bib-0019] Buduneli, N. , & Kinane, D. F. (2011). Host‐derived diagnostic markers related to soft tissue destruction and bone degradation in periodontitis. Journal of Clinical Periodontology, 38, 85–105. 10.1111/j.1600-051x.2010.01670.x 21323706

[cre2192-bib-0050] Cardoso, C. R. , Garlet, G. P. , Crippa, G. E. , Rosa, A. L. , Júnior, W. M. , Rossi, M. A. , & Silva, J. S. (2009). Evidence of the presence of T helper type 17 cells in chronic lesions of human periodontal disease. Oral Microbiology and Immunology, 24(1), 1–6. 10.1111/j.1399-302x.2008.00463.x 19121062

[cre2192-bib-0037] Champagne, C. M. E. , Buchanan, W. , Reddy, M. S. , Preisser, J. S. , Beck, J. D. , & Offenbacher, S. (2003). Potential for gingival crevice fluid measures as predictors of risk for periodontal diseases. Periodontology 2000, 31(1), 167–180. 10.1034/j.1600-0757.2003.03110.x 12657001

[cre2192-bib-0054] Dai, J. , Bi, L. , Lin, J. , & Qi, F. (2017). Evaluation of interleukin‐10 producing CD19+ B cells in human gingival tissue. Archives of Oral Biology, 84, 112–117. 10.1016/j.archoralbio.2017.09.009 28985543

[cre2192-bib-0024] Dar, H. Y. , Azam, Z. , Anupam, R. , Mondal, R. K. , & Srivastava, R. K. (2018). Osteoimmunology: The Nexus between bone and immune system. Frontiers in Bioscience, 23, 464–492. 10.2741/4600 28930556

[cre2192-bib-0003] Díaz‐Zúñiga, J. , Melgar‐Rodríguez, S. , Rojas, L. , Alvarez, C. , Monasterio, G. , Carvajal, P. , & Vernal, R. (2017). Increased levels of the T‐helper 22‐associated cytokine (interleukin‐22) and transcription factor (aryl hydrocarbon receptor) in patients with periodontitis are associated with osteoclast resorptive activity and severity of the disease. Journal of Periodontal Research, 52(5), 893–902. 10.1111/jre.12461 28393368

[cre2192-bib-0057] Dutzan, N. , Gamonal, J. , Silva, A. , Sanz, M. , & Vernal, R. (2009). Over‐expression of forkhead box P3 and its association with receptor activator of nuclear factor‐κB ligand, interleukin (IL)‐17, IL‐10 and transforming growth factor‐β during the progression of chronic periodontitis. Journal of Clinical Periodontology, 36(5), 396–403. 10.1111/j.1600-051x.2009.01390.x 19419438

[cre2192-bib-0041] Dutzan, N. , Vernal, R. , Hernandez, M. , Dezerega, A. , Rivera, O. , Silva, N. , … Gamonal, J. (2009). Levels of interferon‐gamma and transcription factor T‐Bet in progressive periodontal lesions in patients with chronic periodontitis. Journal of Periodontology, 80(2), 290–296. 10.1902/jop.2009.080287 19186970

[cre2192-bib-0042] Gao, Y. , Grassi, F. , Ryan, M. R. , Terauchi, M. , Page, K. , Yang, X. , … Pacifici, R. (2007). IFN‐γ stimulates osteoclast formation and bone loss in vivo via antigen‐driven T cell activation. Journal of Clinical Investigation, 117(1), 122–132. 10.1172/jci30074 PMC1697800 17173138

[cre2192-bib-0025] Garlet, G. P. (2010). Destructive and protective roles of cytokines in periodontitis: A re‐appraisal from host defense and tissue destruction viewpoints. Journal of Dental Research, 89(12), 1349–1363. 10.1177/0022034510376402 20739705

[cre2192-bib-0043] Garlet, G. P. , Cardoso, C. R. B. , Campanelli, A. P. , Garlet, T. P. , Avila‐Campos, M. J. , Cunha, F. Q. , & Silva, J. S. (2008). The essential role of IFN‐γ in the control of lethal *Aggregatibacter actinomycetemcomitans* infection in mice. Microbes and Infection, 10(5), 489–496. 10.1016/j.micinf.2008.01.010 18403243

[cre2192-bib-0047] Garlet, G. P. , Martins, W. , Fonseca, B. A. L. , Ferreira, B. R. , & Silva, J. S. (2004). Matrix metalloproteinases, their physiological inhibitors and osteoclast factors are differentially regulated by the cytokine profile in human periodontal disease. Journal of Clinical Periodontology, 31(8), 671–679. 10.1111/j.1600-051x.2004.00545.x 15257746

[cre2192-bib-0004] Ghighi, M. , Llorens, A. , Baroukh, B. , Chaussain, C. , Bouchard, P. , & Gosset, M. (2018). Differences between inflammatory and catabolic mediators of peri‐implantitis and periodontitis lesions following initial mechanical therapy: An exploratory study. Journal of Periodontal Research, 53(1), 29–39. 10.1111/jre.12483 28898426

[cre2192-bib-0010] He, X.‐T. , Li, X. , Xia, Y. , Yin, Y. , Wu, R.‐X. , Sun, H.‐H. , & Chen, F.‐M. (2019). Building capacity for macrophage modulation and stem cell recruitment in high‐stiffness hydrogels for complex periodontal regeneration: Experimental studies *in vitro* and in rats. Acta Biomaterialia, 88, 162–180. 10.1016/j.actbio.2019.02.004 30735811

[cre2192-bib-0005] Hienz, S. A. , Paliwal, S. , & Ivanovski, S. (2015). Mechanisms of bone resorption in periodontitis. Journal of Immunology Research, 2015(10), 1–10. 10.1155/2015/615486 PMC4433701 26065002

[cre2192-bib-0058] Hong, M. H. , Williams, H. , Jin, C. H. , & Pike, J. W. (2000). The inhibitory effect of interleukin‐10 on mouse osteoclast formation involves novel tyrosine‐phosphorylated proteins. Journal of Bone and Mineral Research, 15(5), 911–918. 10.1359/jbmr.2000.15.5.911 10804021

[cre2192-bib-0048] Ihn, H. , Yamane, K. , Asano, Y. , Kubo, M. , & Tamaki, K. (2002). IL‐4 up‐regulates the expression of tissue inhibitor of metalloproteinase‐2 in dermal fibroblasts via the p38 mitogen‐activated protein kinase‐dependent pathway. The Journal of Immunology, 168(4), 1895–1902. 10.4049/jimmunol.168.4.1895 11823524

[cre2192-bib-0020] Jäger, A. , & Kuchroo, V. K. (2010). Effector and regulatory T‐cell subsets in autoimmunity and tissue inflammation. Scandinavian Journal of Immunology, 72(3), 173–184. 10.1111/j.1365-3083.2010.02432.x 20696013 PMC3129000

[cre2192-bib-0049] Johnson, R. B. , Wood, N. , & Serio, F. G. (2004). Interleukin‐11 and IL‐17 and the pathogenesis of periodontal disease. Journal of Periodontology, 75(1), 37–43. 10.1902/jop.2004.75.1.37 15025215

[cre2192-bib-0028] Kawai, T. , Matsuyama, T. , Hosokawa, Y. , Makihira, S. , Seki, M. , Karimbux, N. Y. , … Taubman, M. A. (2006). B and T lymphocytes are the primary sources of RANKL in the bone resorptive lesion of periodontal disease. The American Journal of Pathology, 169(3), 987–998. 10.2353/ajpath.2006.060180 16936272 PMC1698808

[cre2192-bib-0052] Kim, K. W. , Kim, H. R. , Kim, B. M. , Cho, M. L. , & Lee, S. H. (2015). Th17 cytokines regulate osteoclastogenesis in rheumatoid arthritis. The American Journal of Pathology, 185(11), 3011–3024. 10.1016/j.ajpath.2015.07.017 26362732

[cre2192-bib-0014] Knight, E. T. , Liu, J. , Seymour, G. J. , Faggion, C. M. , & Cullinan, M. P. (2016). Risk factors that may modify the innate and adaptive immune responses in periodontal diseases. Periodontology 2000, 71(1), 22–51. 10.1111/prd.12110 27045429

[cre2192-bib-0038] Lamster, I. B. (1992). The host response in gingival crevicular fluid: Potential applications in periodontitis clinical trials. Journal of Periodontology, 63(12 s), 1117–1123. 10.1902/jop.1992.63.12s 1479531

[cre2192-bib-0055] Lee, S.‐Y. , Min, H. K. , Lee, S. H. , Shin, H. J. , Lee, W. Y. , Cho, Y.‐G. , … Park, S.‐H. (2016). IL‐1 receptor antagonist (IL‐1Ra)‐Fc ameliorate autoimmune arthritis by regulation of the Th17 cells/Treg balance and arthrogenic cytokine activation. Immunology Letters, 172, 56–66. 10.1016/j.imlet.2016.02.011 26903194

[cre2192-bib-0030] Liu, Y.‐C. G. , Lerner, U. H. , & Teng, Y.‐T. A. (2010). Cytokine responses against periodontal infection: Protective and destructive roles. Periodontology 2000, 52(1), 163–206. 10.1111/j.1600-0757.2009.00321.x 20017801

[cre2192-bib-0039] Mitani, A. , Niedbala, W. , Fujimura, T. , Mogi, M. , Miyamae, S. , Higuchi, N. , … Noguchi, T. (2015). Increased expression of interleukin (IL)‐35 and IL‐17, but not IL‐27, in gingival tissues with chronic periodontitis. Journal of Periodontology, 86(2), 301–309. 10.1902/jop.2014.140293 25272982

[cre2192-bib-0036] Mogi, M. , & Otogoto, J. (2007). Expression of cathepsin‐K in gingival crevicular fluid of patients with periodontitis. Archives of Oral Biology, 52(9), 894–898. 10.1016/j.archoralbio.2007.01.006 17321485

[cre2192-bib-0027] Mogi, M. , Otogoto, J. , Ota, N. , & Togari, A. (2004). Differential expression of RANKL and osteoprotegerin in gingival crevicular fluid of patients with periodontitis. Journal of Dental Research, 83(2), 166–169. 10.1177/154405910408300216 14742657

[cre2192-bib-0044] Monasterio, G. , Castillo, F. , Ibarra, J. P. , Guevara, J. , Rojas, L. , Alvarez, C. , … Vernal, R. (2018). Alveolar bone resorption and Th1/Th17‐associated immune response triggered during *Aggregatibacter actinomycetemcomitans*‐induced experimental periodontitis are serotype‐dependent. Journal of Periodontology, 89(10), 1249–1261. 10.1002/jper.17-0563 30030845

[cre2192-bib-0016] Murphy, K. M. , & Reiner, S. L. (2002). The lineage decisions of helper T cells. Nature Reviews Immunology, 2(12), 933–944. 10.1038/nri954 12461566

[cre2192-bib-0011] Ni, C. , Zhou, J. , Kong, N. , Bian, T. , Zhang, Y. , Huang, X. , … Yan, F. (2019). Gold nanoparticles modulate the crosstalk between macrophages and periodontal ligament cells for periodontitis treatment. Biomaterials, 206, 115–132. 10.1016/j.biomaterials.2019.03.039 30933774

[cre2192-bib-0031] Oliveira, T. , Figueiredo, C. A. , Brito, C. , Stavroullakis, A. , Ferreira, A. C. , Nogueira‐Filho, G. , & Prakki, A. (2015). *Allium cepa* L. and quercetin inhibit RANKL/*Porphyromonas* gingivalis LPS‐induced osteoclastogenesis by downregulating NF‐κB signaling pathway. Evidence‐Based Complementary and Alternative Medicine, 2015, 1–11. 10.1155/2015/704781 PMC4529940 26273314

[cre2192-bib-0032] Ozaki, Y. , Koide, M. , Furuya, Y. , Ninomiya, T. , Yasuda, H. , Nakamura, M. , … Udagawa, N. (2017). Treatment of OPG‐deficient mice with WP9QY, a RANKL‐binding peptide, recovers alveolar bone loss by suppressing osteoclastogenesis and enhancing osteoblastogenesis. PLoS One, 12(9), e0184904. 10.1371/journal.pone.0184904 28937990 PMC5609750

[cre2192-bib-0006] Pan, S. , Yang, D. , Zhang, J. , Zhang, Z. , Zhang, H. , Liu, X. , & Li, C. (2018). Temporal expression of interleukin‐22, interleukin‐22 receptor 1 and interleukin‐22‐binding protein during experimental periodontitis in rats. Journal of Periodontal Research, 53(2), 250–257. 10.1111/jre.12512 29080226

[cre2192-bib-0002] Papapanou, P. N. , & Susin, C. (2017). Periodontitis epidemiology: Is periodontitis under‐recognized, over‐diagnosed, or both? Periodontology 2000, 75(1), 45–51. 10.1111/prd.12200 28758302

[cre2192-bib-0045] Pestka, S. , Krause, C. D. , Sarkar, D. , Walter, M. R. , Shi, Y. , & Fisher, P. B. (2004). Interleukin‐10 and related cytokines and receptors. Annual Review of Immunology, 22(1), 929–979. 10.1146/annurev.immunol.22.012703.104622 15032600

[cre2192-bib-0046] Pradeep, A. R. , Roopa, Y. , & Swati, P. P. (2008). Interleukin‐4, a T‐helper 2 cell cytokine, is associated with the remission of periodontal disease. Journal of Periodontal Research, 43(6), 712–716. 10.1111/j.1600-0765.2007.01079.x 18624944

[cre2192-bib-0017] Queiroz‐Junior, C. M. , Silva, M. J. , Correa, J. D. , Madeira, M. F. , Garlet, T. P. , Garlet, G. P. , … da Silva, T. A. (2010). A controversial role for IL‐12 in immune response and bone resorption at apical periodontal sites. Clin Dev Immunol, 2010, 1–8. 10.1155/2010/327417 PMC3042606 21350602

[cre2192-bib-0018] Repeke, C. E. , Ferreira, S. B. , Vieira, A. E. , Silveira, E. M. , Avila‐Campos, M. J. , da Silva, J. S. , … Garlet, G. P. (2011). Dose‐response met‐RANTES treatment of experimental periodontitis: A narrow edge between the disease severity attenuation and infection control. PLoS One, 6(7), e22526. 10.1371/journal.pone.0022526 21799885 PMC3140528

[cre2192-bib-0053] Ribeiro, F. V. , Pino, D. S. , Franck, F. C. , Benatti, B. B. , Tenenbaum, H. , Davies, J. E. , … Casati, M. Z. (2017). Resveratrol inhibits periodontitis‐related bone loss in rats subjected to cigarette smoke inhalation. Journal of Periodontology, 88(8), 788–798. 10.1902/jop.2017.170025 28492360

[cre2192-bib-0056] Sakaguchi, S. (2004). Naturally arising CD4+ regulatory T cells for immunologic self‐tolerance and negative control of immune responses. Annual Review of Immunology, 22, 531–562. 10.1146/annurev.immunol.21.120601.141122 15032588

[cre2192-bib-0034] Sakellari, D. , Menti, S. , & Konstantinidis, A. (2008). Free soluble receptor activator of nuclear factor‐κB ligand in gingival crevicular fluid correlates with distinct pathogens in periodontitis patients. Journal of Clinical Periodontology, 35(11), 938–943. 10.1111/j.1600-051x.2008.01314.x 18988315

[cre2192-bib-0015] Silva, N. , Abusleme, L. , Bravo, D. , Dutzan, N. , Garcia‐Sesnich, J. , Vernal, R. , … Gamonal, J. (2015). Host response mechanisms in periodontal diseases. Journal of Applied Oral Science, 23(3), 329–355. 10.1590/1678-775720140259 26221929 PMC4510669

[cre2192-bib-0040] Stadler, A. F. , Angst, P. D. M. , Arce, R. M. , Gomes, S. C. , Oppermann, R. V. , & Susin, C. (2016). Gingival crevicular fluid levels of cytokines/chemokines in chronic periodontitis: A meta‐analysis. Journal of Clinical Periodontology, 43(9), 727–745. 10.1111/jcpe.12557 27027257

[cre2192-bib-0007] Takayanagi, H. (2005). Inflammatory bone destruction and osteoimmunology. Journal of Periodontal Research, 40(4), 287–293. 10.1111/j.1600-0765.2005.00814.x 15966905

[cre2192-bib-0021] Takayanagi, H. , Ogasawara, K. , Hida, S. , Chiba, T. , Murata, S. , Sato, K. , … Taniguchi, T. (2000). T‐cell‐mediated regulation of osteoclastogenesis by signalling cross‐talk between RANKL and IFN‐γ. Nature, 408(6812), 600–605. 10.1038/35046102 11117749

[cre2192-bib-0029] Teitelbaum, S. L. , & Ross, F. P. (2003). Genetic regulation of osteoclast development and function. Nature Reviews Genetics, 4(8), 638–649. 10.1038/nrg1122 12897775

[cre2192-bib-0060] Wang, L. , Guan, N. , Jin, Y. , Lin, X. , & Gao, H. (2015). Subcutaneous vaccination with *Porphyromonas* gingivalis ameliorates periodontitis by modulating Th17/Treg imbalance in a murine model. International Immunopharmacology, 25(1), 65–73. 10.1016/j.intimp.2015.01.007 25604387

[cre2192-bib-0012] Wu, R.‐X. , He, X.‐T. , Zhu, J.‐H. , Yin, Y. , Li, X. , Liu, X. , & Chen, F.‐M. (2019). Modulating macrophage responses to promote tissue regeneration by changing the formulation of bone extracellular matrix from filler particles to gel bioscaffolds. Materials Science and Engineering: C, 101, 330–340. 10.1016/j.msec.2019.03.107 31029326

[cre2192-bib-0059] Xiao, L. , Zhu, L. , Yang, S. , Lei, D. , Xiao, Y. , & Peng, B. (2015). Different correlation of sphingosine‐1‐phosphate receptor 1 with receptor activator of nuclear factor kappa B ligand and regulatory T cells in rat periapical lesions. Journal of Endodontics, 41(4), 479–486. 10.1016/j.joen.2014.10.010 25492490

[cre2192-bib-0013] Yu, Y. , Wu, R.‐X. , Yin, Y. , & Chen, F.‐M. (2016). Directing immunomodulation using biomaterials for endogenous regeneration. Journal of Materials Chemistry B, 4(4), 569–584. 10.1039/c5tb02199e 32262939

[cre2192-bib-0051] Zheng, Y. , Sun, L. , Jiang, T. , Zhang, D. , He, D. , & Nie, H. (2014). TNFα promotes Th17 cell differentiation through IL‐6 and IL‐1β produced by monocytes in rheumatoid arthritis. Journal of Immunology Research, 2014(385352), 1–12. 10.1155/2014/385352 PMC4243768 25436214

[cre2192-bib-0008] Zhou, L. N. , Bi, C. S. , Gao, L. N. , An, Y. , Chen, F. , & Chen, F. M. (2019). Macrophage polarization in human gingival tissue in response to periodontal disease. Oral Diseases, 25(1), 265–273. 10.1111/odi.12983 30285304

[cre2192-bib-0023] Zhu, J. , & Paul, W. E. (2010). Heterogeneity and plasticity of T helper cells. Cell Research, 20(1), 4–12. 10.1038/cr.2009.138 20010916 PMC3494736

